# Crowdsourced educational resource to distinguish Translational Science (TS) from Translational Research (TR)

**DOI:** 10.1017/cts.2026.10747

**Published:** 2026-05-06

**Authors:** Nathaniel Hafer, Karen Cielo, Paul Duguid, Mendy Dunn, Maryam Gholami, Kristi Holmes, Joe Hunt, Cathleen Kane, Patricia Moussatche, Christy Navarro, Brittney Jackson Patterson, Alfred Vitale, Jennifer Croker

**Affiliations:** 1 Center for Clinical and Translational Science, University of Massachusetts Chan Medical School, Worcester, MA, USA; 2 Office of Research, University of Illinois Chicago, Chicago, IL, USA; 3 Translational Research Institute, University of Arkansas for Medical Sciences, Little Rock, AR, USA; 4 Clinical and Translational Research, Clinical and Translational Science Institute, University of Florida, Gainesville, FL, USA; 5 University of California San Diego Altman Clinical and Translational Research Institute, San Diego, CA, USA; 6 Northwestern University Department: Feinberg School of Medicine, Galter Health Sciences Library & Learning Center, Department of Preventive Medicine, Division of Health and Biomedical Informatics, Chicago, IL, USA; 7 Indiana Clinical and Translational Science Institute, Indiana University, Bloomington, IN, USA; 8 Clinical and Translational Science Center, NYU Langone Health, New York, NY, USA; 9 Stanford’s Center for Clinical and Translational Research and Education, Stanford University, Palo Alto, CA, USA; 10 University of California Davis, Sacramento, CA, USA; 11 Wake Forest School of Medicine, Winston-Salem, NC, USA; 12 Clinical & Translational Science Institute, Mayo Clinic, Rochester, MN, USA; 13 The University of Alabama at Birmingham Heersink School of Medicine, Birmingham, AL, USA

**Keywords:** Translational research, translational science, pilot funding, education, ctsa

The goal of the Clinical and Translational Science Awards (CTSA) Consortium, in partnership with the National Center for Advancing Translational Science (NCATS), is to bring more treatments to all people more quickly. An emerging focus for NCATS is translational science (TS), the field that addresses longstanding scientific and operational challenges through innovations that transform the way research is conducted, making it faster, more efficient, and more impactful [1, 2]. Another key activity is translational research (TR), which is the endeavor to traverse a particular step of the translational process for a particular target or disease [3]. Over the past few years there has been debate and confusion about the definition of these terms [4].

To address these concerns, a session was held during the CTSA Administrators Consortium Committee’s annual meeting on November 13, 2024, to discuss ongoing efforts to better define TS and TR. Drawing on concepts of nominal group technique (5), Administrators summarized the current understanding, brainstormed about best practices, and discussed what kind of concrete products could be developed to clarify the differences between these concepts. The group split into approximately 10 tables and deliberated on what (1) educational resources, (2) written guides, (3) coaching and navigation, (4) directed tools and templates, and (5) other deliverables that may be helpful to elevating a shared understanding. Ideas were captured on sticky notes and compiled by session organizers.

After the annual meeting, a work group formed to analyze the information and to propose a useful product for the consortium. This analysis illustrated significant efforts across hubs in developing learning instruments and signaled the opportunity to coordinate such assets and make them discoverable. The group decided to create a database of educational materials that describe TS, translational research, and the differences between the two. The team briefed the CTSA Administrators on this effort and asked them to list educational resources and activities at their hubs related to TS and TR. An editable document was shared by email and discussed at the December 2024 and February 2025 Administrators meetings. Individuals were asked to provide a brief description of the asset, its purpose, relevant URL, utility or effectiveness to date, CTSA institution, contact person, and contact email.

Appreciating that other consortium groups were doing complementary work, sub-committee members leveraged existing roles and relationships to liaise with Evaluators, Workforce Development, and Quality Assurance/Quality Control (QA/QC) groups. This outreach was critical to avoid duplicate effort, allowing the team to discover what others were doing and how we could coordinate efforts. The crowdsourcing process was repeated with these groups between May and July 2025, which enhanced coverage and saturation of the list of resources and activities.

The initial response included 87 entries from 31 CTSA Hubs. Nine hubs were in the East, eight in the South, and seven each in the Midwest and West. Individual hubs contributed 1–14 resources to the database. A full list of participating institutions is found in column G of the Google sheet [7]. Members of the committee reviewed and curated the entries for relevance, accuracy, duplication, and completeness. Committee members performed targeted outreach to hubs to complete or correct information. Once complete, committee members reviewed all entries and voted to include or remove items. Entries with a unanimous vote to remove were taken off the list. Committee members discussed and ultimately decided that this phase of work would focus on creating a compendium of educational resources that define TS, TR, and the differences between them. The group decided that a comprehensive evaluation of the content and perceived effectiveness was beyond the scope of this project.

The final work product was deposited on the Coordination, Communication, and Operations Support (CCOS) website for long-term storage. The database and documents folder are also openly accessible via the internet (6–8, Table 1). The CCOS site provides a password protected, stable home for this resource. To help improve the usefulness of this resource, documents available as PDFs were downloaded into a database so they can be found even if their URLs change. In addition, the name of the institution, person, and email address contributing the resource was included with each item. The authors have further disseminated this resource by making presentations to CTSA consortium groups and the upcoming 2026 Association and Clinical and Translational Science meeting.

**Table 1.** Sample content presented in the translational science/Translational research resource repository. The full table is available at https://docs.google.com/spreadsheets/d/1t5q53iCdUM2AXDne7Kk7oTWnogO8hOHj85q91WmwQZI/edit?gid=0#gid=0 Table 1 long description.

Label	Resource 1	Resource 2	Resource 3
**Tag**	Pilot RFA/NOFO	Presentation-video	Presentation-video
**Objective**	Educate pilot applicants about TS	Educate pilot applicants about TS	Video presentation of TS v TR
**Asset/procedure**	Pilot grant RFA	webinar	YouTube Video
**Use/context**	include very specific language about the purpose of these grants; also require applicants discuss how the project addresses scientific or operational barriers to effective and efficient translation, with the goal of developing generalizable principles to accelerate translational research.	Webinar explains application process and our definition of TS	
**URL**	https://www.umassmed.edu/ccts/funding/PPP/	https://www.umassmed.edu/globalassets/ccts/ccts-media/kimberleecantin.mp4	https://www.youtube.com/watch?v = 5ieVoBV0j-A
**Notes on utility/perceived effectiveness**	some people answer this thoughtfully; some people write general, hand-waiving stuff; some people ignore the requirement all togeher	live session had small attendance (∼10); we have pointed people to this resource in future pilot rounds; anecdotal stories say it is helpful	none
**CTSA institution**	UMass	UMass	UAMS TRI
**Contact person**	Nate Hafer	Nate Hafer	Paul Duguid
**Contact email**	nathaniel.hafer@umassmed.edu	nathaniel.hafer@umassmed.edu	pduguid@uams.edu

The authors hope that this resource will provide the clinical and TS community, especially those involved in administration, evaluation, pilot grants, and educational programs, with materials to describe the meaning of TS and TR and how each contributes to scientific advances that improve health for everyone.

## Table 1. Long description

Resources in the research respository are tagged with the following information to help individuals locate relevant material: Tag, Objective, Asset/Procedure, Use/Context, URL, Notes on utility/perceived effectiveness, CTSA institution, Contact person, Contact email

Navigate back to Table 1..
